# Editorial: The epigenetic control of transposable elements in development and in diseases

**DOI:** 10.3389/fgene.2023.1282449

**Published:** 2023-09-04

**Authors:** Jan Padeken, Michelle S. Longworth, Luisa Di Stefano

**Affiliations:** ^1^ Institute of Molecular Biology, Mainz, Germany; ^2^ Cleveland Clinic Lerner Research Institute, Cleveland, OH, United States; ^3^ MCD, Centre de Biologie Intégrative (CBI), Université de Toulouse, CNRS, UPS, Toulouse, France

**Keywords:** transposable elements, chromatin, development, disease, bioinformatics

A considerable part of many eukaryotic genomes consists of repetitive sequences derived from transposable elements (TEs). TEs are genetic elements that have, or once had, the capability to hop around their host genomes. Through their mobility, they threaten host genomic stability; consistently, uncontrolled TE transposition has been linked to infertility and to diseases such as cancer, hemophilia, and neurodegeneration (McCullers and Steiniger, 2017). An increasing body of evidence shows that TE expression is elevated in cancer, and accumulation of TE transcripts and extrachromosomal DNA derived from TEs has been shown to trigger an innate immune response (Cuellar et al., 2017; Zhao et al., 2018; Heinrich et al., 2019; Simon et al., 2019; Ardeljan et al., 2020).

At the same time, however, TEs can positively impact their host genome. They act as drivers of genomic evolution, influence neighbouring gene expression and have been suggested to shape developmental processes. For example, during Zygotic Genome Activation (ZGA) in early mouse embryonic development, the expression of hundreds of mouse endogenous retroviruses (MERV) is induced. It has been shown that MERVs provide alternative promoter sequences that activate zygotic genes (Macfarlan et al., 2012). More recently LINE1 elements were also implicated in pre-implantation embryonic development by inducing changes in chromatin accessibility and gene transcription (Jachowicz et al., 2017; Percharde et al., 2018). Outstanding questions in the field include whether and when the expression of these TEs is essential for development and the identities of the mechanisms by which TE expression is controlled at various stages of development.

In a study published in this special Research Topic, Ansaloni and colleagues, used multiomic approaches to identify a set of evolutionary young LINE1 whose transcripts are present in the oocyte and which are poised for transcription during ZGA. Through a bioinformatic approach, they attribute their transcription to YY1 activity, paving the way to new research aimed at experimentally validating the role of YY1 in their regulation. Importantly, this work reveals a specific, spatial compartmentalization of MERVs transcription from gene-dense genomic regions and LINE1 from intergenic regions that could coordinately influence the progress of ZGA.

Human pluripotent stem cells (hPSCs) are undifferentiated, self-renewing cells that are present very early after conception, and which can give rise to all differentiated adult cell types. In this special Research Topic, the review titled “Transposable Elements in Pluripotent Stem Cells and Human Disease,” by Ma et al., focuses on the various ways in which transposable element (TE) expression and activity affect hPSCs, epigenetic-regulated mechanisms that prevent TE expression in hPSCs, and links between the dysregulation of these mechanisms and human diseases including cancer and neurodegenerative disease. While TEs are largely repressed in most cell types, TE expression and activity is high in hPSCs, and the authors present interesting hypotheses on why this phenomenon occurs. Furthermore, TE sequences influence transcription of both coding and non-coding RNAs in mammalian PSCs, through several different mechanisms, thus impacting PSC homeostasis, pluripotency, and self-renewal. Finally, the authors discuss arguments for the potential contribution of TE sequence fragments in PSCs to tumor development and summarize findings on the epigenetic deregulation of LINE-1 and the endogenous retrovirus, HERV-K in tumor cells and neural cell types present in patients with neurodegenerative disorders.

Another developmental time in which TEs are de-silenced is during germ cell specification when animals epigenetically reprogram their genome to ‘reset’ development. While this is an opportunity for TEs to propagate, it is also a threat to the host progeny’s genome integrity. Taming TE transcription is, therefore, essential for fertility. Eukaryotic genomes have evolved specific molecular machineries to silence TEs transcriptionally and post-transcriptionally. They rely, among others, on the small RNA silencing machinery, DNA transcription factors and chromatin-associated enzymes. One of the chromatin factors involved in TE silencing is the histone demethylase LSD1/KDM1A. Depletion of LSD1 activity induces an increase in the transcription of many TE families in mice (Macfarlan et al., 2011; Ancelin et al., 2016) humans (Sheng et al., 2018), and *Drosophila* (Yang et al., 2019; Lepesant et al., 2020).

In this Research Topic, Ren and colleagues, examine the role of *Caenorhabditis elegans* AMX-1, one of the three worm homologs of the human LSD1 family, in fertility. They report that worms lacking AMX-1 can produce a small number of nonviable eggs. The sterility observed upon AMX-1 depletion is dependent on p53/Cep1 and correlates with an increase in expression of Tc1 and Tc3 transposons but is not dependent on H3K4me2 accumulation. The authors argue that AMX-1 and another LSD1 ortholog, SPR-5, are functionally non-redundant in the germline, and the sterility observed in AMX-1-depleted animals is linked to p53-dependent TE activation. These findings necessitate future studies aimed at validating this hypothesis through genome-wide and genetic approaches.

The repetitive nature and frequent mutations within TE families remain a significant challenge in interpreting genome-wide approaches. Despite a continuous effort in developing new tools that would allow us to reliably map and interpret reads from next-generation sequencing-based approaches, we are still lacking a universal and broadly applicable tool set to interpret transcript levels, chromatin modifications, or protein binding at individual copies of repetitive elements. In this special Research Topic, Emmanuelle Lerat summarizes these challenges and provides an overview of the current approaches. She thereby provides a concise list of TE-centric, bioinformatic tools along with brief explanations of their unique features, advantages, and disadvantages.

Many outstanding questions remain to be answered in the field: What are the mechanisms by which TEs are regulated throughout the lifespan of individuals? Which TEs are expressed and when? Do TEs have specific roles in development and in diseases? As research in the field of transposable elements continues to advance, addressing these outstanding questions will provide valuable insights into the roles, regulation, and impacts of TEs on genome evolution, development, and disease.
